# Drone Surveys Do Not Increase Colony-wide Flight Behaviour at Waterbird Nesting Sites, But Sensitivity Varies Among Species

**DOI:** 10.1038/s41598-020-60543-z

**Published:** 2020-03-02

**Authors:** Jared R. Barr, M. Clay Green, Stephen J. DeMaso, Thomas B. Hardy

**Affiliations:** 1 Department of Biology, Texas State University, 601 University Drive, San Marcos, Texas 78666 USA; 2 U.S. Fish and Wildlife Service, Gulf Coast Joint Venture, 700 Cajundome Boulevard, Lafayette, Louisiana 70506 USA; 3 Present Address: California Department of Fish and Wildlife, 3883 Ruffin Road, San Diego, California 92123 USA

**Keywords:** Behavioural ecology, Conservation biology

## Abstract

The popularity of using unmanned aerial vehicles (UAVs) to survey colonial waterbirds has increased in the past decade, but disturbance associated with this bourgeoning technology requires further study. Disturbance was investigated by conducting aerial surveys with a consumer-grade quadcopter (DJI Phantom 3), while concurrently recording behavioural reactions on video. Surveys of mixed-species waterbird colonies (1-6 species per colony) were flown in horizontal transects at heights of 122, 91, 61, and 46 m, which is a typical range for collecting aerial imagery and producing high-resolution mosaicked orthophotos of nesting bird sites. An upper limit of 122 m was used due to local regulations prohibiting higher-altitude flights without federal authorization. Behavioural reactions were tallied every minute and a disturbance score was calculated for each sampling period. When compared to control periods, we found no evidence that colony-wide escape (i.e., flight) behaviour increased during drone flights, at any altitude flown. However, disturbance score increased significantly by 53% for surveys at 46 m. Some species were more sensitive to surveys than others. Laughing Gulls, in particular, exhibited a significant (125%) increase in escape behaviour for surveys at 91 m. Our results indicate when used in a capacity to gather high-resolution imagery for estimating breeding pairs, UAV surveys affected some species more than others, but severe reactions did not appear to increase for mixed-species colonies as a whole. Further study on safe operating thresholds is essential, especially at local and regional scales.

## Introduction

Colonial waterbirds can be exposed to both recreational and investigator disturbance during the breeding season. They congregate in groups to nest, such that a single disturbance event can have a negative impact on multiple individual birds. This is problematic for waterbird researchers and managers, given the importance of conducting long-term monitoring programs on these important bioindicators of ecosystem health^1–3^. Disturbance can be defined as any human activity that alters the behaviour or physiology of one or more individuals of a breeding colony^4^. This can be elicited by colony intrusion, including by that of vehicles, recreationists, or researchers. Thus, research and monitoring schemes that mitigate the negative impacts of disturbance should be prioritized. Colonial waterbirds have historically been surveyed during the breeding season using ground-based counts, manned aircraft, or a combination of the two.

Investigator intrusions are those that involve walking near or through a nesting colony. Ground counts are just one form of investigator intrusion, which involves walking within or around a colony site and counting nests to get an estimate of adult breeding pairs. Although considered a precise and accurate surveying technique, ground counts within a breeding colony can cause significant disturbance, potentially leading to nest abandonment, greater exposure to predation, nest failure from exposure to the elements, and spilled nest contents^5–7^. The manifestation of adverse effects during or following a disturbance event varies among species and locales. Gulls, terns, skimmers, and alcids, for example, can have lowered reproductive success following investigator disturbance^4,6,8–10^. However, in some instances ground counts do not significantly alter behaviour, reproductive success, or productivity^11^. The magnitude of harmful effects is likely dependent on specific survey methodology, e.g., entering a heronry to count nests is presumably more detrimental than establishing a buffer to count from the perimeter^12^.

Aerial surveys can also disturb nesting colonial waterbirds, sometimes causing severe panic responses, nest abandonment, and delayed return times to the nest^13–15^. It is common for birds to look up, scan more, or remain vigilant during aircraft overflights^16^. Furthermore, prolonged vigilance can divert time and energy away from activities that are needed to increase individual fitness^17,18^. However, some mixed-species nesting colonies are not significantly affected by aerial surveys, behaviourally or reproductively^19,20^. Certain species, e.g., Least Tern (*Sternula antillarum*), Common Tern (*Sterna hirundo*), Gull-billed Tern (*Gelochelidon nilotica*), and Black Skimmer (*Rynchops niger*), do not react noticeably to manned aircraft, suggesting that overflights do not detract from their incubation behaviour^21^. This is not always the case, as Adélie Penguins (*Pygoscelis adeliae*) are more prone to abandon nests after helicopter and fixed-wing aircraft surveys^15^.

Unmanned aerial vehicles (UAVs) have recently garnered attention for surveying wildlife. They have been used to survey a variety of avifauna, including Common Terns^22^, Black-headed Gulls (*Chroicocephalus ridibundus*)^23^, cliff-nesting seabirds^24^, and other species that lend themselves well to aerial photography. UAVs provide benefits that make them appealing for surveying wildlife, such as suitability for fine spatial resolution, researcher safety, transportability, cost, and the ability to easily switch out sensors or payloads^25^. Perhaps the most important potential benefit UAVs provide is a small and quiet platform, which could potentially be less disturbing to wildlife than manned aircraft. Recent studies have found that some species of waterbirds are not significantly disturbed by UAV surveys when flown in horizontal transects^22,26–28^. However, for Adélie and Gentoo Penguins (*Pygoscelis papua*), lower survey altitudes can exacerbate disturbance behaviour even when the UAV is flown horizontally^29^.

Due to the recent popularity and availability of UAVs among researchers, managers, and recreationists, there is a need to continue studying their effects on waterbirds. Knowledge gaps remain, especially regarding methodological best practices and operating thresholds in wildlife research and management. Although recent research has addressed disturbance caused by approach angle^24,26^, hovering^24,30^, and low altitude flyovers^30,31^, few studies have taken into consideration the impact of UAV surveys intended for obtaining high-resolution mosaicked orthophotos of large nesting sites. This type of survey is generally flown in horizontal strip transects and at reasonable heights, which enable photogrammetric ease and image stitching^32^. For waterbirds in particular, few studies have investigated disturbance caused by horizontal UAV surveys at heights greater than 50 m^22,33^, even though it has been suggested that count accuracy does not significantly increase for imagery below 90 m^34^. Safe operating thresholds are needed not only for wildlife researchers, but also for hobbyist pilots. At least in the United States, UAV pilots without certificates of authorization from the Federal Aviation Administration (FAA) must fly below 400 ft. (122 m) in non-restricted airspace. Though the technology is still in its infancy, the FAA forecasts that the hobbyist UAV fleet will grow steadily over the next 5 years, from 1.2 million units sold in 2018 to 1.4 million units by 2023^35^. Further research on the ethical operation of UAVs is necessary for high altitude surveys and flight plans that benefit stereoscopic landscape photography of nesting bird sites.

Our overarching research objective was to investigate disturbance caused by a consumer-grade UAV when surveying nesting waterbirds in a manner appropriate for obtaining high-resolution mosaicked imagery. We hypothesized that when conducting flight missions horizontally and at conservative altitudes (46–122 m), UAV surveys would not severely disturb nesting colonies or increase the overall magnitude of disturbance, and would not severely disturb any nesting species surveyed. We hope to build upon a growing body of research focused on UAV operational thresholds for waterbird species and their nesting colonies as a whole. The findings and conclusions in this article are those of the author(s) and do not necessarily represent the views of the U.S. Fish and Wildlife Service.

## Methods

### Study area

**Figure 1:**
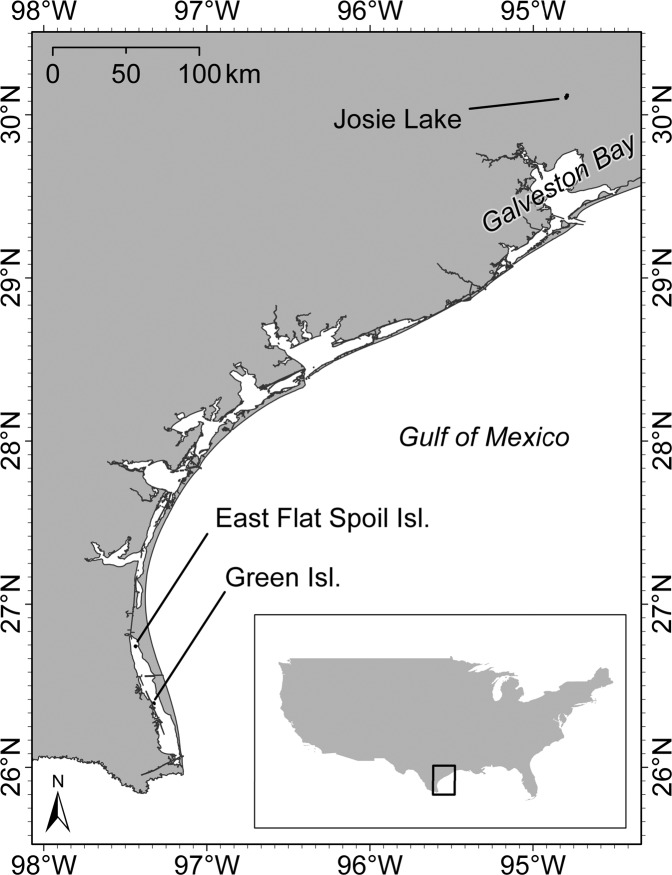
Colonial waterbird nesting sites that were surveyed with an unmanned aerial vehicle (UAV), Texas, USA.

We selected historical nesting sites in Texas to examine disturbance caused by UAV surveys on a mix of colonial waterbird species. Due to variability in nesting strata among colonial nesters, we chose study sites in two distinct regions of Texas: coastal islands along the Gulf Coast and forested wetlands in the Trinity River Basin (Fig. 1). We utilized two colony sites on the Gulf Coast (Green Island and East Flat Spoil Island) and one colony site along the Trinity River (Josie Lake).

Green Island is a 12-ha vegetated island situated in the lower Laguna Madre – a narrow hypersaline coastal lagoon that extends ~185 km from Corpus Christi Bay to the southern tip of Texas^36^. It is a large breeding site for Reddish Egrets (*Egretta rufescens*) and Roseate Spoonbills (*Platelea ajaja*) and is a stop-over site for neotropical migrants^37^. At Green Island, we observed a colony composed of six waterbird species (Table 1). East Flat Spoil Island, located just north of Green, is a 1.2-ha dredge-spoil island with a mix of low-lying vegetation and bare ground habitat. At East Flat Spoil, we observed a colony mostly comprised of five waterbird species. Josie Lake is situated in the lower Trinity River Basin, which has a humid subtropical climate and is predominantly forested^38^. Many waterbird species use Josie Lake to nest and forage, however at the time of our study we only observed four species at this site.

**Table 1:** Unmanned aerial vehicle (UAV) flights over select waterbird colonies in Texas, USA.

	Colony Sites			
	East Flat Spoil	Green Isl.	Josie Lake	
Date	17 May 2016	18 May 2016	24 May 2016	1 June 2017
No. flights	2	2	2	2
Count^a^	1163	1046	230	24
Phase^b^	Eggs	Eggs, nestlings	Eggs, nestlings	Nestlings
Species^c^	Black Skimmer Great Blue Heron Laughing Gull Reddish Egret Royal Tern	Black-crowned Night Heron Great Blue Heron Reddish Egret Roseate Spoonbill Tricolored Heron White Ibis	Anhinga Cattle Egret Great Egret Snowy Egret	Great Egret

^a^Uncorrected adult bird counts from aerial imagery by a single observer.

^b^Qualitative observation of incubation phases at each colony.

^c^Species rarely seen during sample periods and excluded from analysis: Black-crowned Night Heron, Tricolored Heron, Anhinga, and Cattle Egret. Some species could not be identified during sample periods, i.e., small terns and herons far away from video cameras.

### Surveys

We flew surveys horizontal to the ground in a back-and-forth transect pattern at a speed of 3–5 m/s, and did not approach birds from any other angle^26,28^. Strip transects allow for a series of overlapping photos to be captured, which can later be processed into a mosaicked, georeferenced orthophoto^32^. Since a georeferenced aerial photo is what managers would likely use to estimate the number of breeding pairs in a colony, we utilized methods beneficial for this type of data capture. Surveys were conducted with a consumer-grade quadcopter UAV (Phantom 3, DJI, Shenzhen, Guangdong, China) with procedures standardized across all survey missions. The UAV was deployed and brought to altitude roughly 250 m away from the edge of the nesting colony, which was confirmed with a laser range finder (Nikon Aculon IK-714141, Nikon Inc., Tokyo, Japan). The UAV was then flown in a series of sequentially decreasing survey heights of 122, 91, 61, and 46 m above ground level (AGL). We chose these survey heights because it was expected that within this range we could get optimal resolution of aerial photographs with the stock DJI camera (FC300X 1/2.3” CMOS, DJI, Shenzhen, Guangdong, China). Furthermore, an upper limit of 122 m was used due to local regulations prohibiting higher-altitude flights without federal authorization. Each survey was conducted immediately after the other, with the UAV taxied downward to the side of the colony. We conducted flights such that a single battery could be used across all survey transects. Thus, the area surveyed (~1 ha) did not vary greatly among colonies.

Research was conducted in accordance with guidelines, experimental protocols, and regulations of the Texas State University Institutional Animal Care and Use Committee (approved protocol #50). Per FAA regulations, a certified remote pilot operated the UAV below 400 ft. (~122 m) in non-restricted airspace and maintained line of sight during all flights.

### Behavioural observations

Behavioural observations can be used to quantify an animal’s biological response to stimuli^39,40^. We placed video cameras at the periphery of each nesting colony to capture animal behaviour before, during, and after UAV overflights. At Green Island, we positioned cameras in an established bird blind. We placed cameras in areas with an open field of view and selected a random azimuth for adjusting position of the camera lens. If camera frame happened to fall outside of the nesting colony or within the same subset of birds that another camera was focused on, we selected a different azimuth. Camera setups were placed 50–100 m away from the colony because we assumed they would not interfere with the behaviour of nesting waterbirds. Set-back distances vary among waterbird species^36^, so all researchers retreated to ≥250 m away from the colony after video cameras were set to record. It was assumed investigators did not cause additional disturbance, since the farthest set-back distance found in the literature was 178 m for Black Skimmers^41^. We waited ≥20 min following video camera setup to commence surveys, which we assumed would allow birds to return to a baseline level of disturbance prior to UAV surveys. Time stamps on video recordings were synced to the watch of a field observer, who noted the time for each survey. Flight start and end times were delineated by the appearance of the nesting colony in the aircraft’s first-person view mode. Although not exact, this provided consistency for when the UAV was transecting the colony site. Horizontal distance of the UAV to sampled birds was not calculated, given the small and consistent size of surveyed areas.

We used scan sampling, an instantaneous behavioural observation of a group of animals, to capture their behaviour^39^. Since the coloniality of nesting waterbirds is known to have anti-predator advantages, it stands to reason that birds often react to nearby conspecifics or heterospecifics^41^. We chose four behavioural categories *a priori* to assess the extent of disturbance caused by UAV: vigilance, wing flapping, standing at or walking away from the nest, and escape behaviour (i.e., flight). For escape behaviour we tallied birds flying through camera frame as well as flushing from a nest. We reasoned that if UAV surveys caused more birds to initiate escape behaviour, there should be more birds flying within the camera frame during those sample periods. Some species were rarely seen during sample periods so were excluded from analysis (Table 1), and some individuals could not be positively identified to species due to distance from the camera, so they were pooled into two categories, tern *spp*. and heron *spp*. We assumed unidentified terns at East Flat Spoil to be a mix of Gull-billed Terns, Forster’s Terns (*Sterna forsteri*), and Sandwich Terns (*Thalasseus sandvicencis*) based on recent surveys.

One observer (J.R.B.) gathered data from video playback, to eliminate any variation that would arise among multiple observers. Video playback was observed without sound and without the aircraft in frame, so as not to bias observations when a flyover occurred (i.e., J.R.B. was not privy to the occurrence of a survey). Every minute, J.R.B. slowed down video playback to one-third speed, recorded the time, and tallied the behaviour and species of each bird in frame for a total of four seconds. This time period could then later be attributed to its respective treatment group for analysis. We chose a four second sample due to logistical constraints with analysing large groups of birds for extended periods of time. We thought of this time period as a sample of behavioural reactions. If a bird exhibited more than one behaviour during the sample period, the more severe behavioural reaction was recorded (e.g., if a bird was vigilant and then flew from the nest, “escape behaviour” was recorded). Tallies of behavioural reactions were pooled among cameras, since there were two to three cameras used per colony. Logistical constraints prevented us from using three cameras on certain colonies (i.e., vegetation obstructing view). Behavioural responses were assigned weights of 1–4 to differentiate between mild to severe reactions, ranging from vigilance to escape behaviour. Similar methods for indexing disturbance behaviours have been used in other studies^13,26^. We calculated an overall disturbance score with the equation:

1 $$\frac{({V}\,\times \,1)\,+\,({W}\,\times \,2)\,+\,({O}\,\times \,3)\,+\,({E}\,\times \,4)}{N}$$

where *V*, *W*, *O*, and *E* represent the tally of birds that displayed vigilance, wing flapping, off nest standing or walking, and escape behaviour, respectively. *N* represents the total number of individuals during the sample period.

### Statistical analysis

All analyses were conducted in R 3.6.0^42^. We included sample periods that spanned five minutes before and after UAV overflights, so the effect of AGL on colony behaviour could then be compared to a baseline level of disturbance. To test the hypotheses that overall disturbance and escape behaviour (i.e., flight) at the colony-level would not increase, we built and analysed mixed-effects models with R packages lme4 and nlme. These colony-level models were constructed using data in which species were pooled. We used linear models if model assumptions were met (i.e., homoscedasticity and normality), but if there was an indication of non-normality we used binomial generalized linear mixed-effects models with a logit link. Predictor variables included a categorical treatment variable (i.e., pre-flight control, 122 m, 91 m, 61 m, 46 m, and post-flight) and a count index of each colony. We chose to include count index as a predictor in the model because colony size may influence the disturbance to nesting birds^41^. The pre-flight control period was specified as a reference category to which the other levels could then be compared. Since a new cohort of birds were filmed after each overflight, we specified “flight mission” as a random grouping factor, within which we nested treatment. Treatment was included in the random term to avoid issues with pseudo replication.

We investigated the influence of species on both disturbance score and escape behaviour using a similar modelling approach. Using unpooled data, we built linear mixed-effects models that contained an interaction effect between species and treatment. We used sum-to-zero contrasts for the species variable. To ensure independent observations and a proper grouping of species, we nested species within treatment within flight mission as a random grouping factor.

Samples were taken over a temporal scale, so we tested for autocorrelation of residuals using a Ljung-Box test. If temporal autocorrelation was apparent in the model, we incorporated an AR(1) covariance structure using R package nlme. Each full model (i.e., with both random terms for intercept and slope) was tested against a reduced random intercept-only model by likelihood ratio test^43^. We calculated marginal and conditional *R*-square values to assess goodness-of-fit^44^. For linear and generalized linear models, we used restricted maximum likelihood and Laplace approximation for estimating model parameters, respectively. We established the cut-off for statistical significance (α = 0.033) using false discovery rate to correct for multiple comparisons^45^. Parametric bootstrapped 96.7% confidence intervals (*N* = 1,000 iterations) were used to test hypotheses of the fixed effects.

## Results

We had *n* = 190 (species pooled) and *n* = 510 (unpooled) sample observations for 8 UAV overflights conducted in mid-May 2016 and early-June 2017. UAV surveys were conducted between 09:00 and 16:00 hrs on clear to partly cloudy days, with wind conditions ranging between 2.5–23.1 km/hr ($\bar{x}$ = $11.7\pm 8.4\,{\rm{SD}})$. Wind did not hinder or affect the flight patterns of the Phantom quadcopter. Total number of birds in a camera frame ranged from 18–132 ($\bar{x}$ = $56.9\pm 30.8\,{\rm{SD}}$), and the number displaying a behavioural response during all sample periods ranged from 0–29 ($\bar{x}$ = $9.9\pm 6.8\,{\rm{SD}}$). Duration of 122 m surveys ($\bar{x}$ = $170.0\,\sec \pm 59.0\,{\rm{SD}}$), 91 m surveys ($\bar{x}$ = $200.2\,\sec \pm 62.0\,{\rm{SD}}$), 61 m surveys ($\bar{x}$ = $200.0\,\sec \pm 72.7\,{\rm{SD}}$), and 46 m surveys ($\bar{x}$ = $304.0\,\sec \pm 125.9\,{\rm{SD}}$) never exceeded eight minutes.

**Figure 2:**
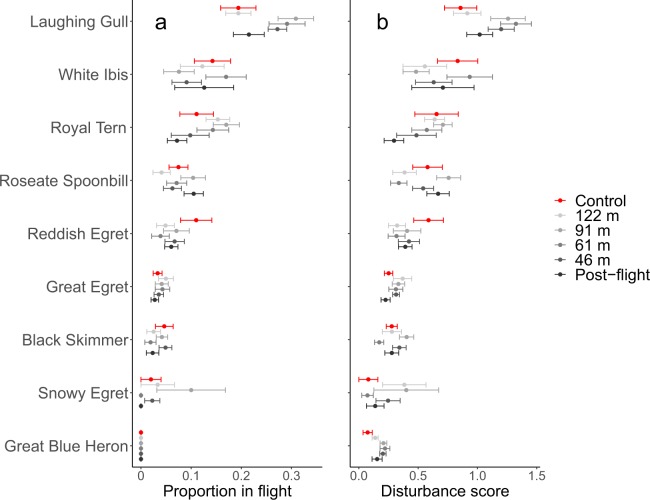
Species-specific mean and SE of (**a**) the proportion of birds that exhibited a flight response and (**b**) their disturbance scores, for unmanned aerial vehicle (UAV) surveys in Texas, USA. Surveys were conducted at 122, 91, 61, and 46 m above ground level. Disturbance scores were calculated by weighting four behavioural categories and creating a proportion based on the total number of birds: ([*V* × 1] + [*W* × 2] + [*O* × 3] + [*E* × 4])/*N*, where *V, W, O*, and *E* represent the tally of birds that displayed vigilance, wing flapping, off nest standing or walking, and escape behaviour, respectively. *N* represents the total number of individuals in the sample.

There were no “dread” flights exhibited by nesting birds during UAV surveys. “Dreads” are when all or most individuals in a colony flush from the nest, circle, and then land^46^. Exploratory analysis revealed that the magnitude of behavioural responses varied among species, with some showing an increase in escape behaviour for specific survey AGLs (Fig. 2). For example, the proportion of Laughing Gulls exhibiting escape behaviour initially increased for surveys at 91 m and then decreased for lower surveys, but other species like Great Blue Herons did not exhibit escape responses during any sample periods.

### Colony-wide disturbance

Surveys conducted at 46 m significantly increased colony-wide (i.e., species pooled) disturbance scores by 52.8%; surveys at 122, 91, and 61 m had no effect when compared to the control (Table 2). 91 m surveys increased colony disturbance by 43.1%, but this was not significant after bootstrapping confidence intervals. For every one-unit change in colony size, there was a significant 0.0003 increase in disturbance score (96.7% CI [0.0002, 0.0004]). The full model for colony disturbance score contained predictors of survey treatment and colony size. The reduced random intercepts-only model was more parsimonious (${\chi }_{\,4}^{2}$ = 0.36, *P* = 0.98) and showed no evidence of temporal autocorrelation (${\chi }_{\,1}^{2}$ = 1.68, *P* = 0.19). This model fits the data well, with the fixed effects of treatment and colony size explaining 49% of model variance (${R}_{\,m}^{2}$ = 0.49, ${R}_{\,c}^{2}$ = 0.63). The random grouping factor of treatment nested within flight mission explained 24% of variance in the model, but there was little variation across missions (ICC = 0.26, SD = 0.08).

**Table 2:** Models showing the effect of unmanned aerial vehicle (UAV) survey height treatments (122, 91, 61, and 46 m) on colony-wide disturbance score and flush response.

Model^a^		Output				
Structure	Predictors	β	SE	Diff.^b^	Lower CI^c^	Upper CI
Disturbance score = treatment + colony size + random(flight mission/treatment)	Control	0.195	0.055		0.081	0.307
	122 m	0.009	0.039	4.6%	−0.079	0.092
	91 m	0.084	0.039	43.1%	−0.006	0.174
	61 m	0.030	0.039	15.4%	−0.060	0.122
	46 m^d^	0.103	0.036	52.8%	0.022	0.190
	Post-flight	0.004	0.036	2.1%	−0.084	0.094
	Colony size^d^	0.0003	0.00006		0.0001	0.0004
Proportion flush (logit) = treatment + colony size + random(flight mission/treatment)	Control	0.039^e^	1.474		−4.057	−2.470
	122 m	0.947	1.143	−5.3%	−0.348	0.239
	91 m	1.270	1.139	27.0%	−0.052	0.552
	61 m	1.104	1.145	10.4%	−0.218	0.458
	46 m	1.228	1.131	22.8%	−0.095	0.486
	Post-flight	0.994	1.135	−0.6%	−0.297	0.285
	Colony size^d^	0.002	0.0007		1.001	1.003

^a^We specified mixed-effects models for colony-wide disturbance. A linear model and generalized linear (binomial) model were used for disturbance score and proportion flush, respectively. Disturbance score was calculated by ([*V* × 1] + [*W* × 2] + [*O* × 3] + [*E* × 4])/*N*, where *V*, *W*, *O*, and *E* represent the tally of birds that displayed vigilance, wing flapping, off nest standing or walking, and escape behaviour, respectively. *N* represents the total number of individuals during the sample period.

^b^% difference between the treatment category and the pre-flight control category.

^c^96.7% CIs (parametric bootstrap [*N* = 1,000]) were used to test hypotheses.

^d^Significant predictors.

^e^Coefficients and SEs are exponentiated in the flush model, for ease of interpretability.

Like the disturbance score, the model for assessing escape behaviour contained predictors for survey treatment and colony size. Predictors for survey height indicated slight increases in colony-wide escape behaviour, but none were significant (Table 2). Colony size was the only predictor that had a significant effect on the proportion of birds engaging in escape behaviour, increasing by 0.2% for every one-unit change in colony size (96.7% CI [1.001, 1.003]). We chose the reduced model (${\chi }_{\,4}^{2}$ = 2.66, *P* = 0.62). It was slightly under-dispersed ($\hat{c}$ = 0.84, ${\chi }_{\,181}^{2}$ = 145.8, *P* = 0.97) and had a modest goodness-of-fit (${R}_{\,m}^{2}$ = 0.25, ${R}_{\,c}^{2}$ = 0.42). Temporal autocorrelation was not present in the model (${\chi }_{\,1}^{2}$ = 0.47, *P* = 0.49), so samples were assumed independent. The random grouping of treatment nested within flight mission was highly variable and accounted for 22% of model variance (ICC = 0.22, SD = 0.96).

### Species-specific disturbance

**Figure 3:**
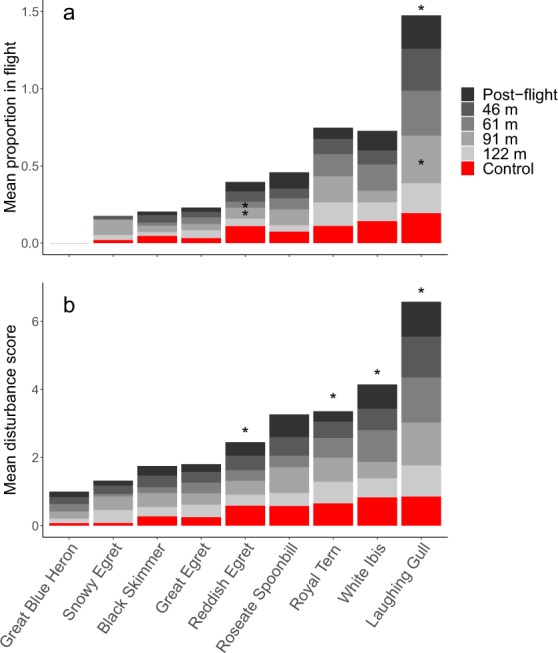
Species-specific mean of (**a**) the proportion of birds that exhibited a flight response and (**b**) their disturbance scores, for unmanned aerial vehicle (UAV) surveys in Texas, USA. Surveys were conducted at 122, 91, 61, and 46 m above ground level. An asterisk (*) above the stacked bar indicates for that species a significant increase across all treatments when compared to the grand mean. An asterisk within the stacked bar indicates for that survey height a significant difference from the pre-flight control period. Disturbance scores were calculated by weighting four behavioural categories and creating a proportion based on the total number of birds: ([*V* × 1] + [*W* × 2] + [*O* × 3] + [*E* × 4])/*N*, where *V*, *W*, *O*, and *E* represent the tally of birds that displayed vigilance, wing flapping, off nest standing or walking, and escape behaviour, respectively. *N* represents the total number of individuals in the sample.

We included an interaction between species and treatment in two models (i.e., proportion flush and disturbance score). Laughing Gulls were the only species to have a significantly higher proportion displaying escape behaviour across all sample periods when compared to the grand mean (96.7% CI [0.057, 0.291], Fig. 3a). On average, the proportion of flushing Laughing Gulls was 135% higher (β = 0.17, SE = 0.05). In addition, 91 m surveys significantly increased Laughing Gull escape behaviour in the colony by 125% when compared to control periods (β = 0.09, SE = 0.04, 96.7% CI [0.002, 0.183], Fig. 3a). 61 m surveys increased their escape behaviour by 126%, but this was not significant after bootstrapping confidence intervals (β = 0.09, SE = 0.04, 96.7% CI [−0.02, 0.21]). Reddish Egrets were the only other species to have significant interaction effects with treatment. When compared to control periods, the proportion of flushing Reddish Egrets decreased significantly by 89.1% and 106.7% for 91 m surveys (β = −0.07, SE = 0.03, 96.7% CI [−0.127, −0.007]) and 61 m surveys (β = −0.08, SE = 0.03, 96.7% CI [−0.145, −0.012]), respectively. The reduced model was more parsimonious (${\chi }_{\,6}^{2}$ = 7.78, *P* = 0.25) and fit the data (${R}_{\,m}^{2}$ = 0.43, ${R}_{\,c}^{2}$ = 0.58). We used an AR(1) covariance structure because the model showed signs of temporal autocorrelation (${\chi }_{\,1}^{2}$ = 17.79, *P* < 0.001).

Of the nine species, four had significantly higher disturbance scores across all survey and non-survey periods when compared to the grand mean (Fig. 3b). These were Laughing Gull (96.7% CI [0.381, 1.374]), Reddish Egret (96.7% CI [0.058, 0.861]), Royal Tern (96.7% CI [0.132, 1.137]), and White Ibis (96.7% CI [0.221, 1.117]). Scores for these species were 90.4%, 3.6%, 37.8%, and 47.3% higher, respectively. There were no significant interactive effects between treatment and species for this model. However, the effect of 61 m surveys increased disturbance score of Laughing Gulls by 99.4% when compared to control periods. This was not significant following confidence interval bootstrapping (β = 0.44, SE = 0.21, 96.7% CI [−0.044, 0.932]). The reduced model was more parsimonious (${\chi }_{\,6}^{2}$ = 4.44, *P* = 0.62) and fit the data (${R}_{\,m}^{2}$ = 0.37, ${R}_{\,c}^{2}$ = 0.51). Temporal autocorrelation was apparent (${\chi }_{\,1}^{2}$= 12.05, *P* < 0.001), so we incorporated an AR(1) covariance structure into the model.

## Discussion

Our objective was to investigate the effect of UAV surveys on waterbird behavioural disturbance. We hypothesized that when conducting flight missions horizontally and at conservative altitudes (46–122 m), UAV surveys would (1) not severely disturb nesting colonies or (2) increase the overall magnitude of disturbance, and (3) would not severely disturb any nesting species surveyed. Colony-wide escape behaviour (i.e., flight) was not influenced by the UAV at any AGL flown, supporting our first hypothesis and suggesting that this platform does not cause severe disturbance to some nesting colonies when flown at reasonable heights for imagery acquisition. However, there were dissimilarities among species, which a colony-wide perspective failed to capture. We found evidence that Laughing Gulls had a higher propensity to initiate an escape response, and thus may be more sensitive to UAV surveys. We failed to support our third hypothesis, given the increased escape behaviour of Laughing Gulls during 91 m surveys. Our other eight study species did not have an increase in severe reactions, which reflects research done on other avian taxa, e.g., Hooded Crows (*Corvus cornix*), Common Terns, Cattle Egret (*Bubulcus ibis*), Snowy Egret, and Glossy Ibis (*Plegadis falcinellus*)^22,31,47^. Recent studies have also found variation among colonial species in their sensitivity to UAVs^24,28^. Some gull species, e.g., Glaucous Gulls (*Larus hyperboreus*) and Iceland Gulls (*Larus glaucoides*), have similar severe reactions^24^. However, one study found that Lesser Black-backed Gulls (*Larus fuscus*) were tolerant of UAV flights even at 15 m AGL, and another found that only 1.25% of observed Black-headed Gulls were in flight during 30–40 m surveys^23,30^. It is generally accepted that tolerance to stimuli can vary among waterbird species, and responses are species-specific^4,41,48^. We reiterate the recommendation from a recent study to conduct baseline tests to determine whether wildlife disperse in response to UAV surveys^24^.

Laughing Gull escape responses increased during 91 m surveys, but not for lower altitudes. This could be explained by birds initially reacting to the UAV, and then rapidly habituating to it. Previous studies suggest that colonial nesting birds can become habituated to horizontal UAV surveys within a short timeframe after initially displaying an increased disturbance response^22,29^. Our methodology could explain the minimal disturbance at 61 and 46 m surveys, since we conducted surveys from high to low AGL in a single flight and the time between surveys did not exceed two minutes. This could have biased our results because individuals that flushed from the nest may not have had enough time to return for lower transects. From a practical standpoint, our results still have value as an indicator of behavioural response thresholds. If the magnitude of severe responses increased at 91 m, then lower flights would generally not be a recommended surveying approach. Operational thresholds such as these can provide helpful guidance to wildlife managers and regulators.

Reddish Egrets had a significant decrease in escape responses during 91 and 61 m surveys when compared to the baseline control. While this is the opposite of what we might expect, there are some possible explanations. First, owing to their status as North America’s rarest heron^49^, not much is known about Reddish Egret disturbance, flight initiation distances, or responses to various stimuli. Perhaps we did not give Reddish Egrets enough time to return to a baseline level of disturbance prior to flights, or the 250 m buffer was too small. The only flight initiation distance found in the literature was 41.14 ± 15.10 m for loafing birds approached by personal watercraft^50^. Another potential source of error that could explain this result is our use of a four-second sample period. We acknowledge that this short time frame is not ideal for capturing animal behaviour because it increases the likelihood that we missed important behavioural cues.

Overall disturbance was measured by distilling multiple types of behavioural reactions into a single score. This score increased significantly for nesting colonies during 46 m UAV surveys, which fails to support our second hypothesis. When taken in context with the model on flush behaviour, this suggests that less severe reactions (e.g., vigilance, walking, standing) increased colony-wide, but escape behaviour did not. Laughing Gull, Reddish Egret, Royal Tern, and White Ibis had significantly higher disturbance scores across all treatments when compared to the grand mean, which indicates their tendency to respond to stimuli more often and display a suite of disturbance behaviours more readily. Thus, it is interesting that survey AGL had no effect on species-specific disturbance scores. We did not investigate survival or reproductive effects caused by these “less severe” behaviours, but, along with escape response, further research is needed on the subject. Prolonged vigilance, for example, has the potential to divert time and energy away from activities that are needed to increase individual fitness in some species^17,18^. Disturbance (flight behaviour or otherwise) did not increase during the five-minute post-flight period in all tests, indicating that birds were quick to resume normal nesting behaviour. However, we did not investigate the duration for which birds were off nest, which would be an indicator of disturbance severity and requires further study.

There are many facets to anthropogenic disturbance that were not included in this study, such as incubation phase, survey frequency, and survey duration. For waterbird species, the egg-laying period and early incubation phases are when birds are most sensitive to disturbance^8,10,51–53^. We did not confirm nesting status of birds in camera frame, but assumed that individuals were actively incubating or brooding. This could potentially have influenced our study because some nonbreeding waterbirds initiate severe reactions to UAV surveys^24,54^. We did not investigate survey frequency, even though high-frequency monitoring schemes can discourage late-nesters from initiating in a colony^52^. Though none of our surveys lasted more than eight minutes, survey duration has the potential to influence behaviour. Our surveys were comparable in duration to that of a previous study, which found UAVs required significantly less time than ground counts^31^. Managers should be aware that flight duration is dependent on area of coverage, AGL, and flight plan.

Although species varied in response to UAV surveys, this type of monitoring may be a favourable alternative to traditional methodology in certain circumstances. With the exception of Laughing Gulls, severe disturbance responses were not increased. Furthermore, “dread flights” did not occur during any survey, and birds were quick to resume normal nesting behaviour following flights. This is promising because flushing from the nest during the breeding season can have negative consequences for waterbirds, as dense colonies are more prone to depredation^55,56^. Coupled with their potential to accurately and precisely count birds^22,34,57^, UAVs may be an auspicious tool for obtaining high-resolution mosaicked orthophotos of colony sites to estimate population size. However, due to their increasing popularity for management, research, and recreation, there is a need to continue investigating the impact of UAVs on wildlife at regional and local scales.

## Data Availability

The datasets generated during and/or analysed during the current study are available from the corresponding author on reasonable request.
